# Neuroprotective effects of Interleukin-1 receptor antagonism after cardiac arrest: an experimental rat study

**DOI:** 10.1007/s00210-026-05135-w

**Published:** 2026-03-16

**Authors:** Bilal Arslan, Fatma Ayaz Yalınkılıç, Adnan Erseçkin, Burhan Beger, Bilal Acar, Utku Batu, Mehmet Mehdi Oğuz, Abdulaziz Yalınkılıç, Orhan Beger, Mehmet Zeki Erdem, Halit Demir

**Affiliations:** 1 Department of Pediatrics, Van Yüzüncü Yıl University Faculty of Medicine, Van, Turkey; 2 Department of Pediatric Surgery, Van Yüzüncü Yıl University Faculty of Medicine, Van, Turkey; 3 Department of Pediatrics, Ağrı Private Lena Hospital, Ağrı, Turkey; 4 Department of Pediatrics, Van Regional Training and Research Hospital, Van, Turkey; 5 Department of Pathology, Van Yüzüncü Yıl University Faculty of Medicine, Van, Turkey; 6 Department of Anatomy, Faculty of Medicine, Gaziantep University, Gaziantep, Turkey; 7 Department of Otorhinolaryngology, Van Yüzüncü Yıl University Faculty of Medicine, Van, Turkey; 8 Department of Biochemistry, Van Yüzüncü Yıl University Faculty of Medicine, Van, Turkey

**Keywords:** Cardiac arrest, Neuroinflammation, IL-1 receptor antagonist, Anakinra, Oxidative stress, Neuroprotection, Experimental model

## Abstract

This study aimed to investigate the histopathological and biochemical neuroprotective effects of IL-1 receptor antagonist (anakinra) administration in an asphyxia-induced cardiac arrest model. A total of 24 Wistar albino rats were randomized into three groups: control, sham (cardiac arrest + saline), and anakinra (cardiac arrest + 50 mg/kg anakinra). Brain tissues were evaluated using a six-parameter semi-quantitative histopathological scoring system, and the composite damage score (CDS) was calculated (0–12). Levels of antioxidant and oxidative markers SOD, CAT, GSH, and MDA were analyzed spectrophotometrically. In the anakinra-treated group, neuronal degeneration, gliosis, and neuropil edema levels were significantly lower compared to the sham group (*p* < 0.01 for all). Although vascular congestion and perivascular edema were observed to be lower in the anakinra group, this reduction was not statistically significant in score analyses compared to the sham group (*p* > 0.05). No significant difference was found between the groups in terms of PMNL infiltration. In biochemical analyses, SOD, CAT, and GSH levels were found to be significantly higher in the anakinra group compared to both the sham and control groups (*p* < 0.001), while MDA levels were significantly lower (*p* < 0.001). These findings indicate that anakinra reduces oxidative stress and significantly alleviates neurohistopathological damage in the experimental model. Anakinra administration significantly reduced short-term hypoxic-ischemic brain damage following cardiac arrest, both in terms of histopathological and oxidative stress parameters. The findings suggest that targeting the IL-1-mediated inflammatory response could be an important neuroprotective approach; however, further studies are needed regarding dose, timing of administration, and long-term neurological function.

**Supplementary Information:**

The online version contains supplementary material available at 10.1007/s00210-026-05135-w.

## Introduction

Even if circulation is restored after cardiac arrest, mortality and serious neurological sequelae rates remain high. Current guidelines emphasize the decisive role of neurological damage in post-cardiac arrest management and recommend systemic approaches such as targeted temperature management, hemodynamic stabilization, adequate ventilation/oxygenation, and multi-organ support. Despite these measures, neurological injury continues to represent a major clinical problem after successful resuscitation (Hirsch et al. 2025).

The pathophysiology of neurological injury following cardiac arrest is a multifactorial process known as post-cardiac arrest syndrome (PCAS), primarily triggered by global cerebral ischemia–reperfusion injury. The systemic inflammatory response associated with PCAS exacerbates neuroinflammation through endothelial dysfunction, disruption of blood–brain barrier integrity, microcirculatory impairment, and microglial and astroglial activation, thereby increasing secondary brain damage. Clinical and experimental data indicate that an intense inflammatory response in the early hours after cardiac arrest is associated with poor neurological outcomes, while processes such as delayed neutrophil infiltration may further contribute to ongoing injury (Dekay et al. 2024).

In this context, the interleukin-1 (IL-1) pathway, a key regulator of the inflammatory cascade, emerges as a targetable component of neural inflammation after cardiac arrest. IL-1 signaling has been linked to neuronal injury, increased blood–brain barrier permeability, glial activation, and elevated pro-inflammatory cytokine production, and cytokine responses are known to be markedly enhanced in tissues following cardiac arrest (Uray et al. 2021).

IL-1 receptor antagonism (IL-1Ra/anakinra) represents a therapeutic strategy with the potential to attenuate secondary brain injury by suppressing IL-1–mediated inflammation. Human studies have demonstrated that IL-1Ra can penetrate the central nervous system and reach the cerebrospinal fluid, while experimental studies have shown reductions in tissue damage and functional deterioration in ischemic injury models. Notably, the neuroprotective effects of IL-1 inhibition with anakinra in ischemia–reperfusion–based stroke models share common pathophysiological mechanisms with post-cardiac arrest brain injury (Clark et al. 2008). In similar ischemia–reperfusion models, IL-1 receptor antagonism has been shown to reduce inflammation and lesion volume; for example, high-dose anakinra decreased infarct size and inflammatory gene expression in transient middle cerebral artery occlusion models (Chaparro‐Cabanillas et al. 2025).

Based on these observations, anakinra appears to be a repositionable candidate for post-resuscitation neuroprotection. Accordingly, the present study aimed to evaluate the effects of anakinra administration during the post-resuscitation period on histopathological damage scores and biochemical oxidative stress parameters in rats subjected to a controlled asphyxia-induced cardiac arrest model. We hypothesized that blockade of the IL-1 pathway would reduce neuroinflammation during the ischemia–reperfusion phase following cardiac arrest, thereby limiting brain injury and improving histopathological and biochemical outcomes.

Cerebral ischemia–reperfusion injury following cardiac arrest is characterized by excitotoxicity, oxidative stress, and a significant inflammatory response. Clinical and experimental studies have shown that increased cytokine release (primarily IL-1β), especially in the early hours of reperfusion, is a key determinant of neurological prognosis (Hirsch et al. 2025; Nolan et al. 2021). Current guidelines for post-cardiac arrest care emphasize the role of the inflammatory response in the progression of neurological damage, in addition to targeted heat management and hemodynamic stabilization (Nolan et al. 2021).

The classical ischemia–reperfusion literature has revealed that IL-1-mediated inflammation is closely associated with disruption of blood–brain barrier integrity, microcirculatory dysfunction, and secondary neuronal damage (Allan et al. 2005; Sobowale et al. 2016). In this context, early targeting of the IL-1 pathway stands out as a potential neuroprotective strategy in the post-resuscitation period.

## Materials and methods

### Ethical approval

This experimental study was conducted in accordance with the approval dated 25/12/2025 and numbered 2025/13–19, obtained from the Van Yüzüncü Yıl University Experimental Animals Local Ethics Committee.

### Experimental animals and housing conditions

In the study, 24 male Wistar Albino rats, weighing 200–250 g and aged 2–3 months, obtained from the Van YYÜ Experimental Medicine Application and Research Center, were used. The experimental animals were housed in rooms with adequate ventilation, at a room temperature of 21–24 °C, with a 12-h light/12-h dark photoperiod, throughout the study period. The rats were kept in standard polycarbonate rat cages. All subjects were given standard pellet rat feed and tap water ad libitum.

### Grouping and randomization

The animals were randomly divided into three experimental groups using a random number table (*n* = 8):

- Group 1— Sham (CA + SF) Cardiac arrest and resuscitation were performed, followed by intraperitoneal administration of 0.5 mL of 0.9% NaCl.
- Group 2— Anakinra: Cardiac arrest and resuscitation were followed by intraperitoneal administration of 50 mg/kg anakinra (Kineret ®).
- Group 3— Control: No surgical of pharmacological procedures were performed.

Randomization and intervention order were determined by the experimental staff, and histopathological analyses were evaluated blindly.

### Anesthesia, intubation, and cardiac arrest model

Ketamine (80 mg/kg, IP) was administered for sedation and analgesia. Oxygen saturation and heart rate monitoring were performed using Nellcor and Mindray pulse oximeters via probes placed in the brachial or femoral regions. For intubation, a 1.2-mm (4 Fr, green) feeding tube was modified under sterile conditions, an intubation adapter was placed at its distal end, and intubation was performed using a 00-gauge laryngoscope blade + stylet.

Following intubation, the tracheal tube was clamped to create controlled respiratory arrest. In this study, cardiac arrest was defined based on non-invasive physiological parameters such as loss of oxygen saturation signal, marked bradycardia, and cessation of spontaneous breathing. Although invasive arterial pressure or electrocardiographic verification was not performed, this approach is commonly used in experimental cardiac arrest models focusing on early hypoxic-ischemic damage. This definition is consistent with previously published asphyxia-induced cardiac arrest models focusing on early hypoxic-ischemic damage (Yu et al. 2023). However, the lack of invasive hemodynamic validation represents a methodological limitation of the study.

Although invasive arterial pressure or ECG validation was not used, the model is consistent with previously validated asphyxia-induced cardiac arrest models. However, this limitation may have led to inter-animal variability in ischemic load.

### Resuscitation and pharmacological application

All interventions after cardiac arrest were performed within the framework of the standard 6-min resuscitation protocol. The sham group received 0.5 mL of saline intraperitoneal (IP), and the anakinra group received 50 mg/kg anakinra IP. In both groups, 0.01 mg/kg adrenaline was administered intraperitoneally every 3 min for a total of three doses during resuscitation. Following the interventions, sacrifice was performed at 6 min, and blood and brain tissue samples were collected for analysis. The anakinra dose of 50 mg/kg is based on the dose range that has been shown to be effective in previous experimental studies of ischemia and traumatic brain injury (Hirik et al. 2018). In addition, the anakinra dose (50 mg/kg) was chosen based on previous experimental studies that reported that it provided adequate passage to the central nervous system and showed anti-inflammatory effects in acute brain injury models (Chaparro‐Cabanillas et al. 2025).

### Histopathological evaluation

Brain tissue samples from all 24 rats (8 rats in each group) were fixed in 10% buffered formaldehyde solution for histopathological examination. After fixation, the tissues were embedded in paraffin blocks after undergoing routine tissue processing procedures. Four-micrometer sections were taken from the paraffin blocks using a microtome. The sections were stained histochemically with hematoxylin–eosin (H&E) for general histopathological evaluation. Evaluations were performed at different magnifications using an Olympus BX53F (Olympus, Tokyo, Japan) light microscope, and photographs of the cases were taken with an Olympus E-330 (Olympus Imaging Corporation, China) camera. Histopathological examination assessed six parameters: neuronal degeneration (cytoplasmic eosinophilia-red neuron, nuclear pyknosis), gliosis/satellitosis, neuropil edema, vascular congestion, perivascular edema, and PMNL infiltration. The parameters were classified as absent (−), mild (+), moderate (+ +), and severe (+ + +), and numbered 0, 1, 2, and 3 to allow for statistical evaluation of the observed changes.

### Biochemical analysis

Blood samples were taken from all rats after sacrifice, and their serum was separated and used for biochemical analyses.

### Superoxide dismutase (SOD) activity

SOD activity was determined by a manual method based on the inhibition of NBT reduction using the xanthine–xanthine oxidase system. The reaction mixture was prepared with a reactive solution containing xanthine, EDTA, NBT, Na₂CO₃, and bovine serum albumin. The reaction was initiated by the addition of xanthine oxidase, and absorbances were measured at 560 nm after the addition of CuCl₂. SOD activity was defined as the enzyme activity that provided 50% inhibition of NBT reduction and was calculated in U/ml.

### Catalase (CAT) activity

Catalase activity was determined according to the Aebi method using hydrogen peroxide as a substrate. The reaction mixture was initiated in a medium containing H₂O₂ and phosphate buffer. The change in absorbance after enzyme addition was measured at 240 nm. Activity was calculated based on the decomposition rate of hydrogen peroxide and expressed in U/L.

### Malondialdehyde (MDA) level

Serum MDA levels were determined using the thiobarbituric acid (TBA) method. Supernatants obtained after protein precipitation and incubation were measured at 532 nm. MDA concentration was calculated in µmol/L using the relevant coefficient.

### Reduced glutathione (GSH) level

Serum GSH levels were determined using the Ellman method. Absorbance measurements were performed at 412 nm after adding DTNB (Ellman reagent) to samples diluted with phosphate buffer. GSH levels were calculated in mmol/g protein using the molar extinction coefficient.

### Statistical analysis

Power analysis was performed using the “G*Power” software package (Version 3.1.9.2). The sample size was determined as 24 (three groups and *n* = 8) with 80% power, 5% margin of error, and an effect size of 0.50. Changes in histopathological parameters across groups were analyzed using the chi-square test. Additionally, changes in the scores of the histopathological parameters of the study groups were analyzed using the Kruskal–Wallis test. After finding significance in the Kruskal–Wallis test, pairwise comparisons between groups were performed using the Mann–Whitney *U* test, and Bonferroni correction was applied (corrected significance level for pairwise comparisons α = 0.017). Comparison of blood parameters in the study groups was analyzed using the Kruskal–Wallis test. Statistical analyses were performed using the SPSS software package (IBM SPSS Version 22.0). A value of “*p* < 0.05” was considered significant.

## Results

### Neuronal degeneration

**Fig. 1:**
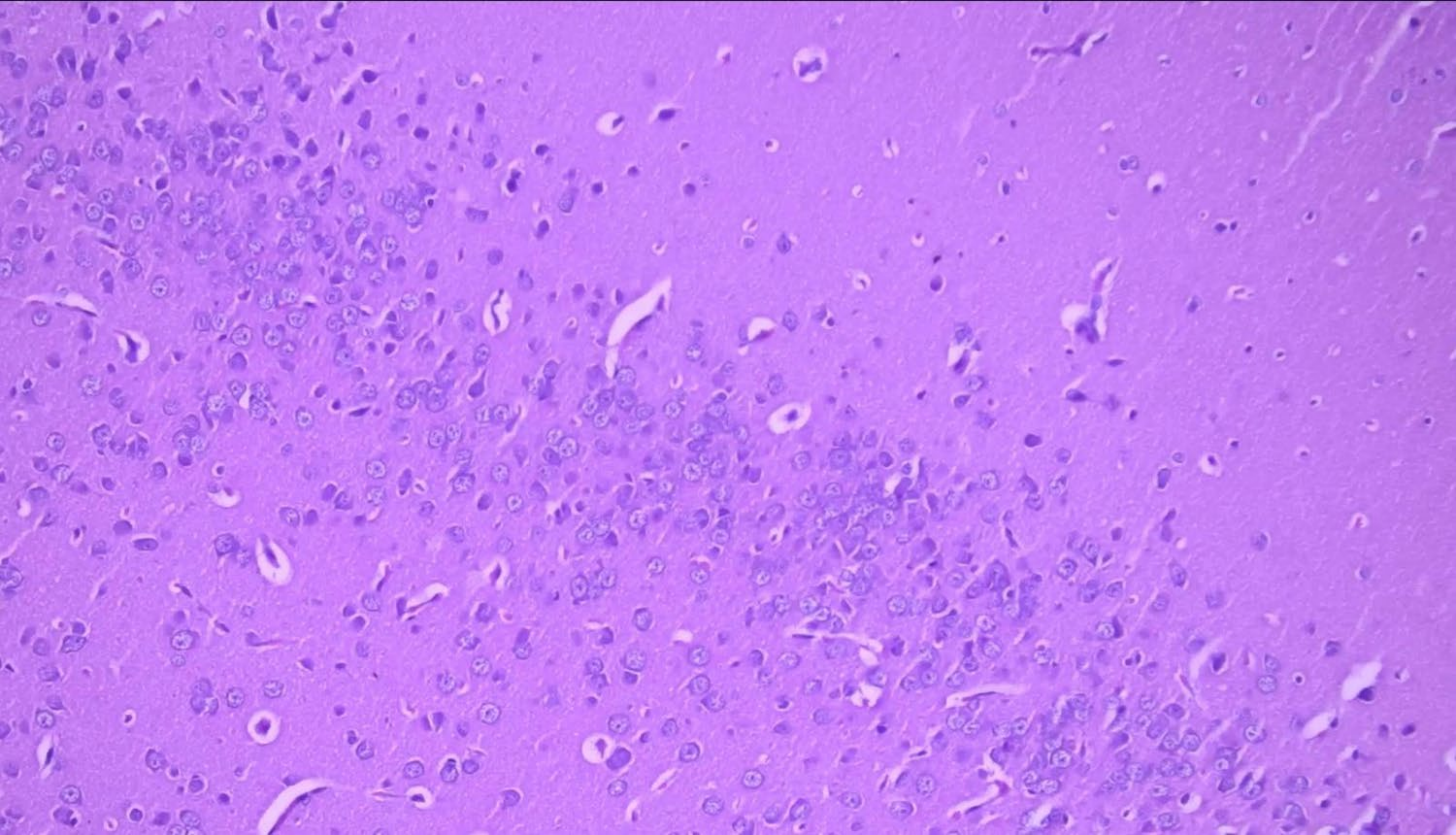
Brain tissue with normal histological characteristics—H&E × 200

The levels of neuronal degeneration differed significantly between the groups in terms of both frequency and scores (chi-square test, *p* < 0.001; Kruskal–Wallis, *H* = 20.81, *p* < 0.001) (Table 1, Fig. 1).

**Table 1:** Evaluation of the histopathological parameters according to study groups (chi-square test)

Neuronal degeneration	0 (−)	1 (+)	2 (+ +)	Total	*p*	Vascular congestion	0 (−)	1 (+)	2 (+ +)	Total	*p*
G1	0 (0%)	2 (25%)	6 (75%)	8	< 0.001	G1	0 (0%)	4 (50%)	4 (50%)	8	< 0.001
G2	0 (0%)	8 (100%)	0 (0%)	8		G2	0 (0%)	7 (87.5%)	1 (12.5%)	8	
G3	8 (100%)	0 (0%)	0 (0%)	8		G3	8 (100%)	0 (0%)	0 (0%)	8	
Total	8	10	6	24		Total	8	11	5	24	
Gliosis	0 (−)	1 (+)	2 (+ +)	Total	*p*	Perivascular edema	0 (−)	1 (+)	2 (+ +)	Total	*p*
G1	1 (12.5%)	7 (87.5%)	-	8	0.002	G1	0 (0%)	2 (25%)	6 (75%)	8	< 0.001
G2	5 (62.5%)	3 (37.5%)	-	8		G2	0 (0%)	6 (75%)	2 (25%)	8	
G3	8 (100%)	0 (0%)	-	8		G3	8 (100%)	0 (0%)	0 (0%)	8	
Total	14	10	-	24		Total	8	8	8	24	
Neuropil edema	0 (−)	1 (+)	2 (+ +)	Total	*p*	PMNL infiltration	0 (−)	1 (+)	2 (+ +)	Total	*p*
G1	0 (0%)	1 (12.5%)	7 (87.5%)	8	< 0.001	G1	8 (100%)	0 (0%)	0 (0%)	8	-
G2	2 (25%)	6 (75%)	0 (0%)	8		G2	8 (100%)	0 (0%)	0 (0%)	8	
G3	8 (100%)	0 (0%)	0 (0%)	8		G3	8 (100%)	0 (0%)	0 (0%)	8	
Total	10	7	7	24		Total	24	0	0	24	

G1, sham group; G2, anakinra group; G3, control group; 0 (−), absent; 1 (+), mild; 2 (+ +), moderate

**Fig. 2:**
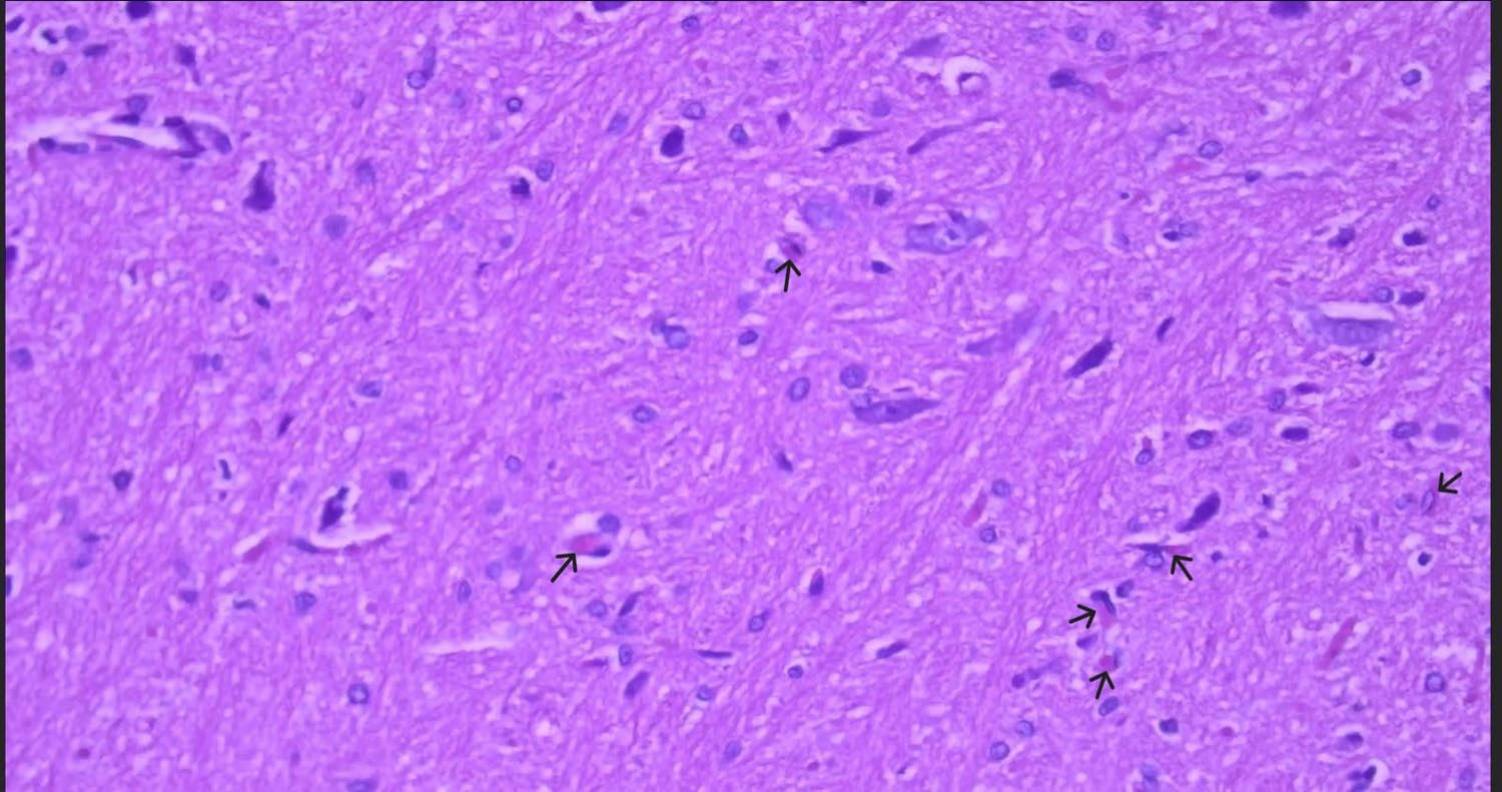
Red neurons (cytoplasmic eosinophilia) in the sham group (unidirectional black arrow)—H&E × 200

In the sham group, mild neuronal degeneration was detected in two rats and moderate neuronal degeneration in six rats. In the anakinra group, only mild neuronal degeneration was observed in all rats (Table 2, Fig. 2).

**Table 2:** Comparison of scores belonging to the histopathological parameters of the study groups (Kruskal–Wallis test)

Parameters	G1	G2	G3	*p*	Effect size (*ε*^2^)
Neuronal degeneration	2 (1–2)^a, b^	1 (1–1)^b^	0	< 0.001	0.90 (large)
Gliosis	1 (0–1)^a, b^	0 (0–1)	0	0.002	0.48 (large)
Neuropil edema	2 (1–2)^a, b^	1 (0–1)^b^	0	< 0.001	0.84 (large)
Vascular congestion	1.50 (1–2)^b^	1 (1–2)^b^	0	< 0.001	0.80 (large)
Perivascular edema	2 (1–2)^b^	1 (1–2)^b^	0	< 0.001	0.79 (large)
PMNL infiltration	0	0	0	1.000	-

Parameters are presented in the table as median (minimum–maximum). G1, sham group; G2, anakinra group; G3, control group

^a^Comparison to G2

^b^Comparison to G3, *p* < 0.05

Effect sizes are presented as epsilon-squared (*ε*^2^) for Kruskal–Wallis tests. Effect size magnitude was interpreted as small (≈0.01), moderate (≈0.06), and large (≥ 0.14)

### In post hoc analyses

- The sham group showed significantly higher neuronal degeneration than the anakinra group (*p* = 0.003).
- The anakinra group was found to be higher than the control group (*p* < 0.001).
- The sham group showed the highest level of degeneration compared to the control group (*p* < 0.001).

These findings indicate that anakinra administration significantly reduced the severity of neuronal degeneration. The effect size of these differences was found to be high (*ε*^2^ = 0.90 for Kruskal–Wallis; *r* = 0.75–0.97 for pairwise comparisons) (Table 2).

### Gliosis

The degrees of gliosis differed significantly between groups (chi-square test, *p* = 0.002; Kruskal–Wallis, *H* = 12.16, *p* = 0.002).

**Fig. 3:**
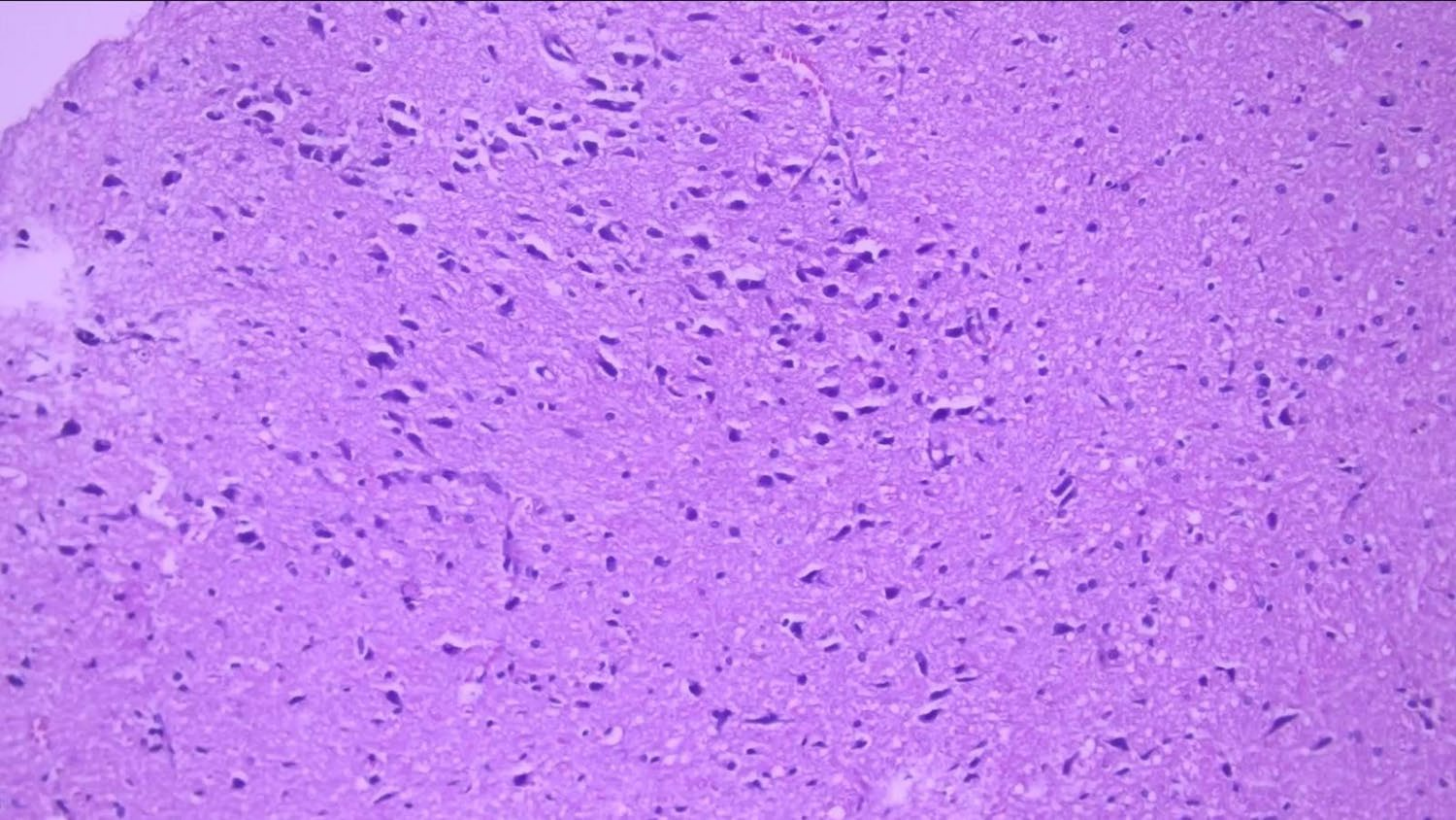
Gliosis and satellitosis in the sham group—H&E × 200

In the sham group, mild gliosis was detected in seven rats, while one rat showed no gliosis (Fig. 3). In the anakinra group, mild gliosis was detected in three rats, and no gliosis was detected in five rats.

In pairwise comparisons, the sham group showed significantly higher gliosis compared to both the anakinra group (*p* = 0.046) and the control group (*p* = 0.001). No significant difference was observed between the anakinra and control groups (*p* = 0.063).

These results reveal that anakinra significantly suppresses the development of gliosis. The observed differences have a moderate-to-high effect size (*ε*^2^ = 0.48; *r* = 0.50–0.86) (Table 2).

### Neuropil edema

**Fig. 4:**
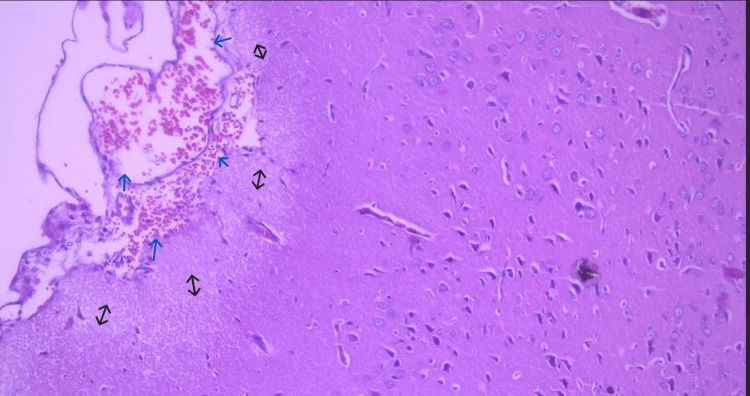
Congestion in the sham group (blue arrow) and edema in the neuropil (double-sided black arrow)—H&E × 200

A significant difference was found between the groups in terms of neuropil edema (chi-square test, *p* < 0.001; Kruskal–Wallis, *H* = 19.56, *p* < 0.001) (Fig. 4).

In the sham group, moderate edema was observed in seven rats and mild edema in one rat. In the anakinra group, mild edema was observed in six rats, and no edema was observed in two rats.

### In post hoc analyses

- Sham group > anakinra group (*p* = 0.001).
- Anakinra group > control group (*p* = 0.003).
- Sham group > control group (*p* < 0.001). Significant differences were found.

Accordingly, anakinra administration significantly reduced both the frequency and severity of neuropil edema. This finding is supported by a high effect size (*ε*^2^ = 0.84; *r* = 0.75–0.94) (Table 2).

### Vascular congestion

**Fig. 5:**
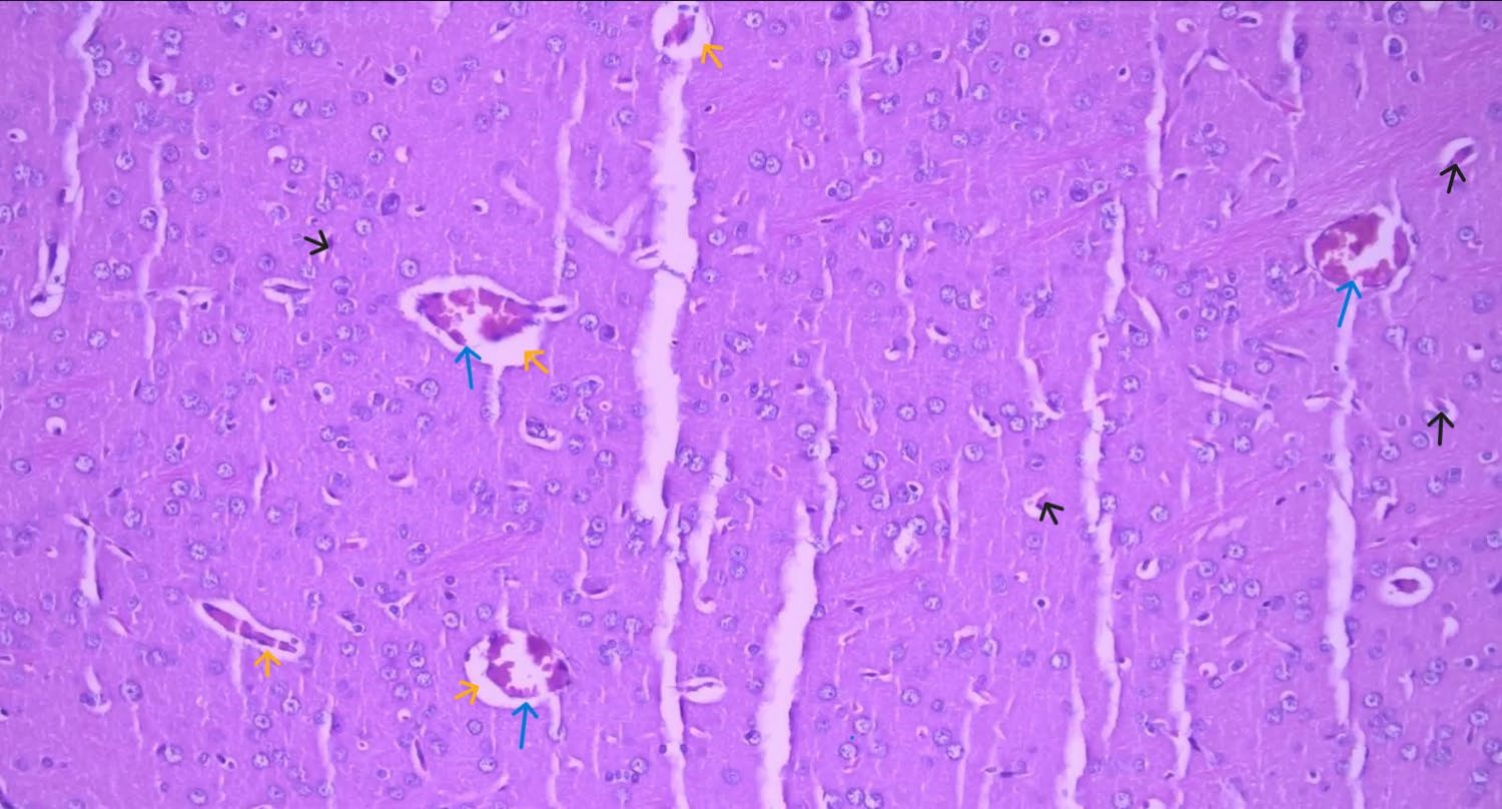
Congestion (blue arrow), perivascular edema (yellow arrow), and red neurons (black arrow) in the sham group—H&E × 200

A significant difference was found between the groups in the assessment of vascular congestion (chi-square test, *p* < 0.001; Kruskal–Wallis, *H* = 18.72, *p* < 0.001) (Fig. 5).

In the sham group, mild congestion was observed in four rats and moderate congestion in four rats. In the anakinra group, mild congestion was observed in seven rats and moderate congestion in one rat.

In score-based analyses, both the sham and anakinra groups showed significantly higher congestion compared to the control group (*p* < 0.001 for both). However, no statistically significant difference was found between the sham and anakinra groups (*p* = 0.117).

These results suggest that anakinra administration did not show a statistically significant reducing effect on vascular congestion (Table 2).

### Perivascular edema

A significant difference was found between the groups in terms of perivascular edema (chi-square test, *p* < 0.001; Kruskal–Wallis, *H* = 18.69, *p* < 0.001).

In the sham group, mild edema was found in two rats and moderate edema in six rats. In the anakinra group, mild edema was observed in six rats and moderate edema in two rats.

Score analyses showed that the sham and anakinra groups had significantly higher values compared to the control group (*p* < 0.001). The difference between the sham and anakinra groups was not statistically significant (*p* = 0.053).

These findings indicate that perivascular edema scores in the anakinra group were not statistically significantly different compared to the sham group (Table 2).

**Fig. 6:**
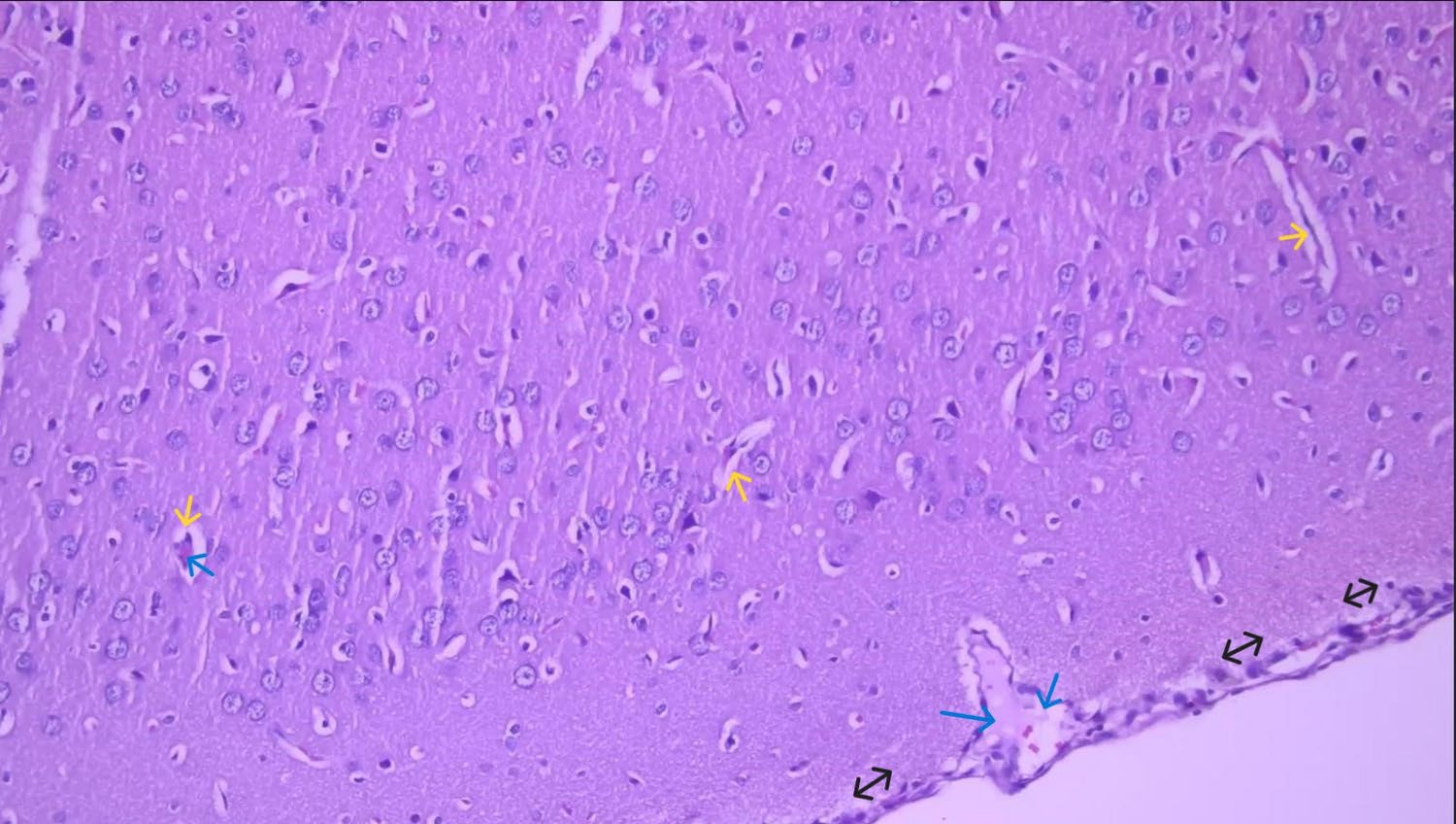
Decrease in congestion (blue arrow), perivascular edema (yellow arrow), neuropil edema (double-sided black arrow), and gliosis in the anakinra group—H&E × 200

Representative improvement in vascular congestion, perivascular edema, neuropil edema, and gliosis in the anakinra group is shown in Fig. 6.

In addition to statistical significance, effect size analyses demonstrated a large magnitude of treatment effect for neuronal degeneration, neuropil edema, and overall histopathological damage scores, indicating that the observed differences were not only statistically significant but also biologically meaningful (Table 2).

### PMNL infiltration

No significant difference was found between the groups in terms of PMNL infiltration (chi-square test, *p* > 0.05; Kruskal–Wallis, *H* = 0.00, *p* = 1.000).

No significant PMNL infiltration was observed in any of the three groups (Table 2).

**Fig. 7:**
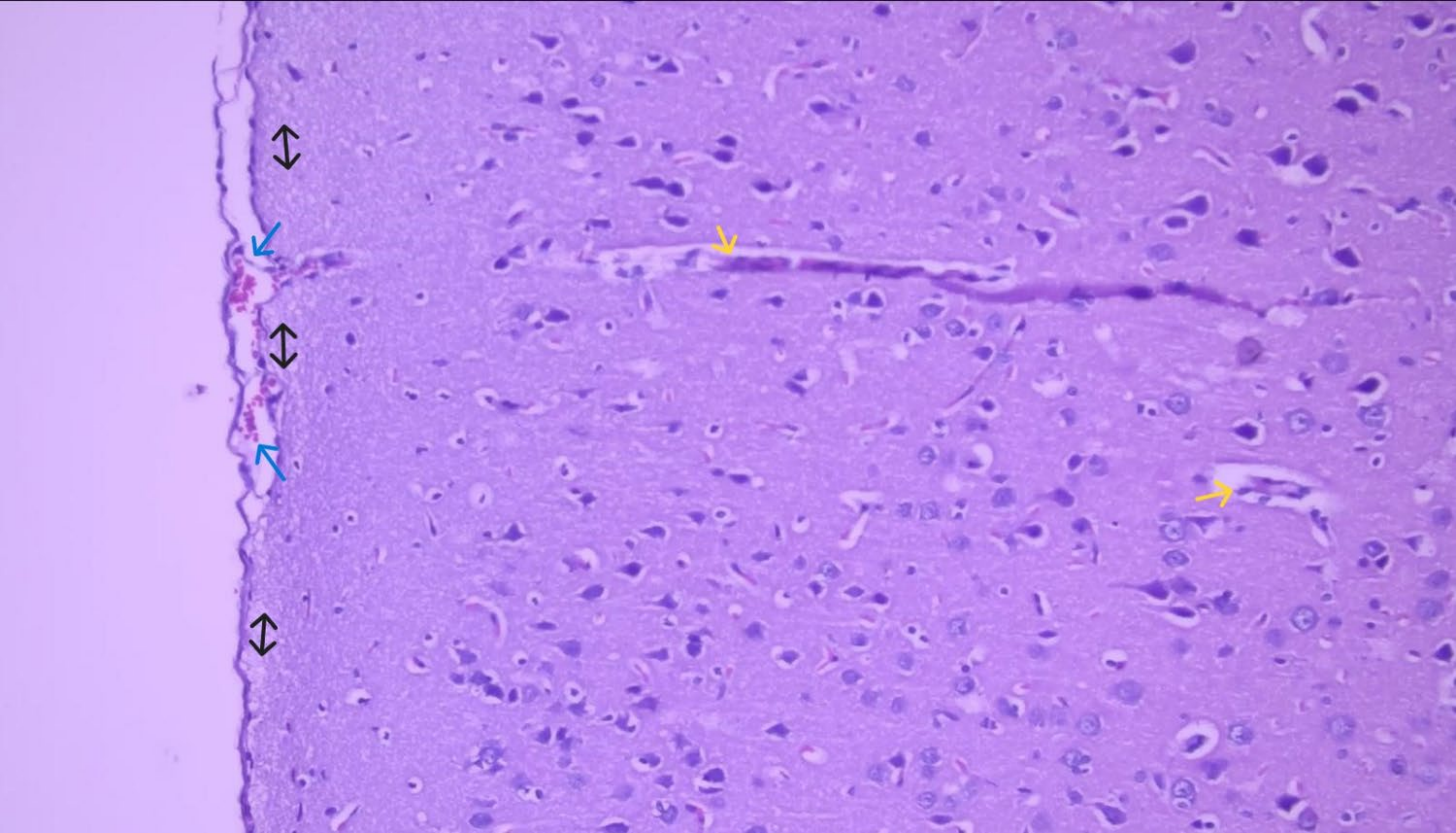
Decrease in congestion (blue arrow), perivascular edema (yellow arrow), neuropil edema (double-sided black arrow), and gliosis in the anakinra group—H&E × 200. All presented histopathological images are representative sections of the respective groups. Histopathological evaluation was performed independently and blindly in each group, and the presented images are consistent with quantitative histopathological scores (Table 2). The number of animals evaluated for each group is indicated in parentheses. All sections were stained with hematoxylin–eosin (H&E) and examined at × 200 magnification

Another representative histopathological section of the anakinra group demonstrating similar improvements is shown in Fig. 7.

### Biochemical findings (SOD, CAT, GSH, MDA)

Significant differences were found between the groups in terms of antioxidant and oxidative stress markers (Kruskal–Wallis, *p* < 0.001) (Table 3).

- SOD, CAT, and GSH levels were found to be significantly higher in the anakinra group than in both the sham and control groups.
- In addition, these parameters were significantly higher in the control group compared to the sham group.
- MDA level was significantly lower in the anakinra group compared to both the sham and control groups.
- MDA level in the sham group was found to be lower than in the control group.

**Table 3:** Comparison of blood parameters of the study groups (Kruskal–Wallis test)

Parameters	G1	G2	G3	*p*	Effect size (*ε*^2^)
SOD	(median, 0.44; range, 0.41–0.47)^a;b^	(median, 1.10; range, 1.02–1.16)^b^	(median, 0.70; range, 0.62–0.75)	< 0.001	0.88 (large)
CAT	(median, 0.25; range, 0.21–0.26)^a;b^	(median, 0.57; range, 0.52–0.62)^b^	(median, 0.29; range, 0.25–0.32)	< 0.001	0.83 (large)
GSH	(median, 0.17; range, 0.15–0.19)^a;b^	(median, 0.43; range, 0.39–0.46)^b^	(median, 0.23; range, 0.21–0.29)	< 0.001	0.88 (large)
MDA	(median, 1.69; range, 1.58–1.77)^a;b^	(median, 1.15; range, 1.11–1.18)^b^	(median, 2.15; range, 2.09–2.19)	< 0.001	0.88 (large)

G1, sham group; G2, anakinra group; G3, control group

^a^Comparison to G2

^b^Comparison to G3, *p* < 0.05

Effect sizes are presented as epsilon-squared (*ε*^2^) for Kruskal–Wallis tests. Effect size magnitude was interpreted as small (≈0.01), moderate (≈0.06), and large (≥ 0.14)

These findings indicate that anakinra treatment significantly reduces oxidative stress and strengthens the antioxidant defense system.

Similarly, effect size measures for oxidative stress parameters revealed a strong treatment effect of anakinra on antioxidant enzyme levels and lipid peroxidation markers, further supporting the robustness of the biochemical findings (Table 3).

## Discussion

### Originality of the study and early-stage focused design

The originality of our study lies in the evaluation of early biochemical and histopathological parameters in rats that underwent early resuscitation after experimental hypoxic brain injury. Thus, we identified the early positive effects of anakinra rather than its late effects. Recent experimental studies have shown that secondary brain damage is regulated by increased cytokine release, oxidative stress, and microglial response within the first hours (Simon et al. 2017; Campbell et al. 2010). In addition, several studies indicate that early interventions are more decisive in determining subsequent neuroprotective effects (Simon et al. 2017; Zheng et al. 2022).

### IL-1 pathway and early neuroinflammation

It has been reported that IL-1β expression rapidly increases during the first hours after central nervous system injury, triggering microglial activation, blood–brain barrier disruption, glutamate excitotoxicity, and mitochondrial dysfunction (Allan et al. 2005). Furthermore, recent studies have demonstrated that IL-1 antagonism not only suppresses inflammation but also provides direct neuronal protection (Sobowale et al. 2016). Clinical studies and international guidelines have shown that systemic and central inflammatory responses developing in the early post-cardiac arrest period are closely associated with long-term neurological outcomes (Marasini and Jia 2024; Nolan et al. 2021). In particular, the rapid increase in IL-1β levels during the early reperfusion phase has been associated with increased blood–brain barrier permeability, cerebral edema, and microcirculatory impairment (Allan et al. 2005; Sobowale et al. 2016).

Classical experimental studies on reperfusion injury indicate that inflammation and oxidative stress are key determinants of secondary brain damage (Simon et al. 2017; Bergold 2016). In this context, the early histopathological improvement and reduction in oxidative stress parameters observed with anakinra in our study are consistent with the existing clinical and experimental literature. Our findings support IL-1 antagonism as a potential neuroprotective target in the early post-resuscitation period; however, further studies are required to determine whether these effects translate into long-term functional outcomes.

### Consistency of histopathological findings with the literature

In our study, the significant early reduction in neuronal degeneration, gliosis, and neuropil edema in the anakinra-treated group demonstrates that suppression of the IL-1–mediated inflammatory process provides tissue-level neuroprotection. Recent experimental stroke and traumatic brain injury studies have similarly shown reductions in cortical neuron loss, suppression of microglial proliferation, and preservation of white matter integrity (Lindblad et al. 2023; Galea et al. 2011; Bergold 2016).

Notably, in the study by Galea et al., IL-1 antagonism reached the central nervous system within 45 min and exerted neuroprotective effects; our findings further support both the consistency of the literature and the effectiveness of anakinra at earlier stages (Galea et al. 2011).

### Suppression of early oxidative stress

In our study, early increases in SOD, CAT, and GSH levels, along with decreased MDA levels, indicate that anakinra can effectively control reperfusion-induced oxidative damage during the early phase. Recent studies emphasize oxidative stress as one of the two principal pathophysiological mechanisms progressing alongside inflammation in secondary brain injury (Marasini and Jia 2024).

Experimental studies have reported that anakinra preserves mitochondrial membrane potential, reduces lipid peroxidation, and enhances antioxidant gene expression, findings that are consistent with the results of our study (Pariano et al. 2021).

### Early resuscitation and clinical implications

The implementation of early resuscitation in our model enhances the clinical relevance of our findings. Current intensive care and neuroresuscitation guidelines emphasize that post-resuscitation inflammatory syndrome developing within the first hours after cardiac arrest and severe trauma is a key determinant of long-term neurological prognosis (Nolan et al. 2021).

Rapid increases in IL-1β and TNF-α levels after reperfusion have been reported to cause capillary leakage, cerebral edema, and microcirculatory dysfunction. The observed reduction in histopathological brain damage following early anakinra administration in our study suggests that the drug may provide potential clinical benefit, particularly within the early therapeutic time window after resuscitation.

### Limited response of microvascular findings

The limited statistical effect observed for vascular congestion and perivascular edema parameters suggests that these findings may primarily reflect early hemodynamic instability, increased capillary hydrostatic pressure, and endothelial dysfunction. Although experimental studies indicate that IL-1 blockade strongly affects neuronal and glial injury and may also influence microvascular morphology, the lack of statistical significance in our study may be related to the early study period and the limited sample size. These findings warrant confirmation in larger-scale experimental studies (Hasturk et al. 2015).

### General assessment

Our study demonstrates that early administration of anakinra significantly reduces histopathological brain damage by concurrently suppressing inflammatory responses and oxidative stress. These findings suggest that anakinra may represent a potential neuroprotective treatment during the early phase of clinical conditions such as traumatic brain injury, hypoxic–ischemic encephalopathy, and post-cardiac arrest syndrome. However, further experimental and randomized clinical trials are necessary to determine optimal dosing, duration of treatment, repeated dosing requirements, and long-term functional outcomes.

**Fig. 8:**
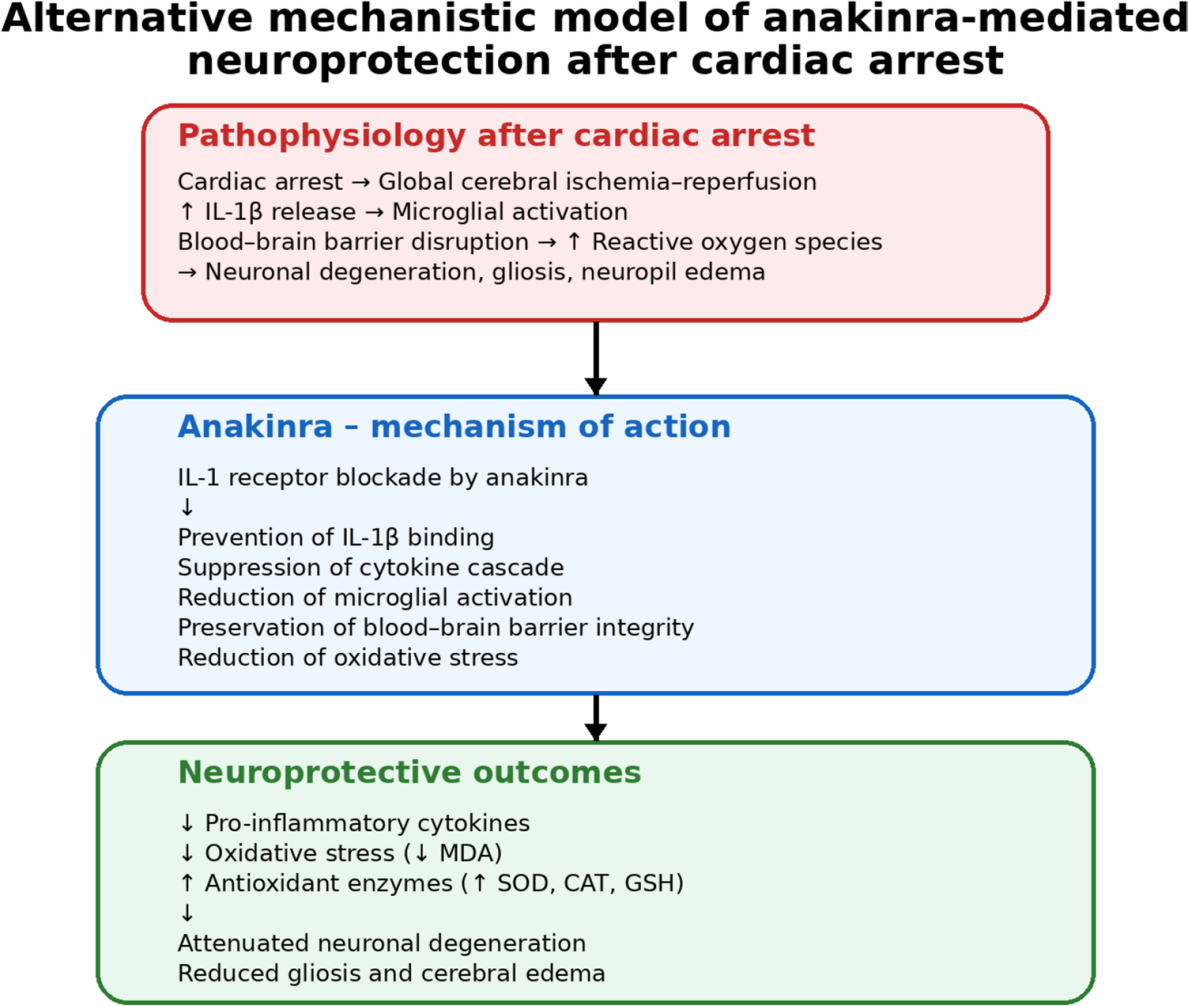
Mechanistic model of anakinra-mediated neuroprotection after cardiac arrest. Cardiac arrest induces global cerebral ischemia–reperfusion injury, leading to increased IL-1β release, microglial activation, blood–brain barrier disruption, and oxidative stress, resulting in neuronal degeneration, gliosis, and neuropil edema. Anakinra exerts its neuroprotective effects by blocking IL-1 receptors, suppressing the cytokine cascade, reducing microglial activation and oxidative stress, increasing antioxidant enzyme activity (SOD, CAT, GSH), and ultimately attenuating histopathological brain injury

Based on our histopathological and biochemical findings, we propose a mechanistic model illustrating the neuroprotective effects of anakinra following cardiac arrest (Fig. 8).

### Study limitations

This study has several limitations that need to be considered. First, the experimental design model focused only on early post-resuscitation histopathological and biochemical outcomes, and long-term neurological or functional assessments (e.g., neurological deficit scores, behavioral or cognitive tests after 7–14 days) were not performed. Therefore, although anakinra demonstrated significant early neuroprotective effects at the tissue and biochemical levels, it cannot be directly inferred that these findings translate into long-term, sustained functional neurological recovery. Future studies incorporating standardized neurological scoring systems and longer follow-up periods are needed.

Second, anakinra was evaluated only for a single dose (50 mg/kg) and a single administration time. Since dose–response relationships and optimal therapeutic ranges are critical for clinical translation, additional studies investigating different dosing regimens, repeated dosing strategies, and delayed administration protocols are needed.

Third, confirmation of cardiac arrest relied on non-invasive physiological criteria (apnea, loss of oxygen saturation signal, and severe bradycardia) rather than invasive arterial pressure monitoring or electrocardiographic validation. While this approach is commonly used in asphyxia-induced cardiac arrest models focusing on early hypoxic-ischemic damage, the lack of invasive validation may have led to inter-animal variability in ischemic load, which should be considered when interpreting the results.

Fourth, the study primarily assessed neuronal and glial damage markers, without direct microvascular or blood–brain barrier integrity assessments (e.g., endothelial markers or permeability tests). This may partially explain the limited statistical effect observed in vascular congestion and perivascular edema parameters.

Finally, detailed hemodynamic parameters and systemic physiological variables such as potential effects on other organs were not comprehensively evaluated, and therefore, systemic contributions to the observed neuroprotective effects cannot be entirely ruled out.

## Supplementary Information

Below is the link to the electronic supplementary material.

ESM 1 (PDF 149 KB)

## Data Availability

The datasets generated and/or analyzed during the current study are available from the corresponding author on reasonable request.
