# Supplementary material for: Neuroprotective effects of Interleukin-1 receptor antagonism after cardiac arrest: an experimental rat study
Source: Naunyn Schmiedebergs Arch Pharmacol. 2026 Mar 16;399(8):12573–84. doi: 10.1007/s00210-026-05135-w (PMC13269372; doi:10.1007/s00210-026-05135-w)
Supplement: Supplementary file 1 — (PDF 149 KB) [file 210_2026_5135_MOESM1_ESM.pdf]

	**VAN YÜHADYEK**<br>**VAN YÜZÜNCÜ YIL ÜNİVERSİTESİ**<br>**Hayvan Deneyleri Yerel Etik Kurulu**

ARAŞTIRMA KESİN SONUÇ ONAY BELGESİ

VAN YUZUNCUYILUNIVERSITY (TURKEY)
ANIMAL RESEARCHES LOCAL ETHIC COMMITTEE
RESEARCH FINAL REPORT APPROVAL CERTIFICATE

**AraştırmanınAdı**<br><br>*Research Title*	Deneysel Kardiyak Arrest Sonrası Gelişen Asfikside IL-1 İnhibitörünün (Anakinra) Etkinliğinin Araştırılması<br><br>Investigation of the Efficacy of IL-1 Inhibitor (Anakinra) in Asphyxia Developing After Experimental Cardiac Arrest	
**Araştırıcı(lar)**<br>*Investigator(s)*	**Yürütücü** / *Chief investigator :*	Dr. Öğr. Üyesi Bilal ARSLAN
	**YardımcıAraştırıcı(lar)** / *Co-investigator(s):*	Dr. Öğr. Üyesi Abdulaziz Yalınkılıç<br>Prof. Dr. Burhan Beger<br>Dr. Öğr. Üyesi Fatma Ayaz Yalınkılıç
AraştırmanınBaşlamaTarihi / *Research Starting Date:* 14.04.2025		
AraştırmanınBitişTarihi / *Research Completion Date:* 17.11.2025		
**ProjeSüresi** / *Total Time of Project:7 ay*		
**Proje No** / *Project Number:* 2025/03-08		
**AraştırmayıDestekleyenKuruluş (varsa)**/ *Funding institution(s) (if available):*		
**Destek Şekli ve Miktarı** / *Type and amount of funding:*		

Karar:
Yukarıda bilgileri verilen araştırma projesinin kesin sonuç raporu Van Yüzüncü Yıl Üniversitesi Hayvan Deneyleri Yerel Etik Kurulu'nun 25/12/2025 tarih ve 2025/13-19 sayılı kararı ile kabul edilmiştir.
Decision:
Final report of the research project detailed above was approved by Van Yuzuncu Yil University Animal Researches Local Ethic Committee in the session held on 25/12/2025 (decision number2025/13-19)

	**BAŞKAN /CHAIR**<br><br>Prof. Dr. Semiha DEDE	
**ÜYE**<br><br>Prof. Dr. N. Tuğba BİNGÖL	**ÜYE**<br><br>Prof. Dr. Sıddık KESKİN	**ÜYE**<br><br>Prof. Dr. Atilla DURMUŞ
**ÜYE**<br><br>Prof. Dr. Nalan ÖZDAL	**ÜYE**<br><br>Prof. Dr. Yıldıray BAŞBUĞAN	**ÜYE**<br><br>Prof. Dr. Canser Yılmaz DEMİR
**ÜYE**<br><br>Prof. Dr. Abdulahad DOĞAN	**ÜYE**<br><br>Doç. Dr. Ferda KARAKUŞ	**ÜYE**<br><br>Doç. Dr. Dicle ALTINDAL
**ÜYE**<br><br>Doç. Dr. Şükrü ÖNALAN	**ÜYE**<br><br>Dr. Öğr. Üyesi Meryem ÇALİŞİR	**ÜYE**<br><br>Vet. Hek. Ferhat İŞİK
**ÜYE**<br><br>Vet. Hek. Ümit IŞIK	**ÜYE**<br><br>Yasin DEMİRHAN	
